# Analysis of MicroRNA Profile Alterations in Extracellular Vesicles From Mesenchymal Stromal Cells Overexpressing Stem Cell Factor

**DOI:** 10.3389/fcell.2021.754025

**Published:** 2021-11-15

**Authors:** Ekaterina Zubkova, Evgeniy Evtushenko, Irina Beloglazova, German Osmak, Phillip Koshkin, Alexander Moschenko, Mikhail Menshikov, Yelena Parfyonova

**Affiliations:** ^1^Federal State Budgetary Institution (FSBI), “National Medical Research Center of Cardiology,” Ministry of Health of the Russian Federation, Moscow, Russia; ^2^Faculty of Chemistry, Lomonosov Moscow State University, Moscow, Russia; ^3^Genomed Ltd., Moscow, Russia; ^4^Federal Center of Brain Research and Neurotechnologies, Federal Medical Biological Agency, Moscow, Russia

**Keywords:** mesenchymal stromal cells, extracellular vesicles, adeno-associated viral vectors, miRNA, stress

## Abstract

Mesenchymal stem/stromal cells (MSCs) represent a promising tool to treat cardiovascular diseases. One mode of action through which MSCs exert their protective effects is secretion of extracellular vesicles (EVs). Recently, we demonstrated that rat adipose-derived MSC-overexpressing stem cell factor (SCF) can induce endogenous regenerative processes and improve cardiac function. In the present work, we isolated EVs from intact, GFP- or SCF-overexpressing rat MSC and analyzed microarray datasets of their miRNA cargo. We uncovered a total of 95 differentially expressed miRNAs. We did not observe significant differences between EVs from GFP-MSC and SCF-MSC that may indicate intrinsic changes in MSC after viral transduction. About 80 miRNAs were downregulated in EVs from both SCF- or GFP-MSC. We assembled the miRNA-based network and found several nodes of target genes among which Vim Sept3 and Vsnl1 are involved in regulation of cellular migration that is consistent with our previous EVs data. Topological analyses of the network also revealed that among the downregulated miRNA-rno-miRNA-128-3p that regulates plenty of targets is presumably associated with chemokine signaling pathways. Overall, our data suggest that genetic modification of MSC has a great impact on their miRNA composition and provide novel insights into the regulatory networks underlying EV effects.

## Introduction

During the last decade, enormous research in the field of extracellular vesicles (EV) has been carried out. Previously, synthetic liposomes and nanovesicles have been used as pharmaceutical vehicles for drug delivery. However, there are still questions concerning their biocompatibility and toxicity of their lipid membranes. Intrinsic EVs could potentially overcome some of the limitations of synthetic liposomes and provide options as carriers of therapeutic molecules, specifically miRNA. Mesenchymal stromal cells (MSC) are considered as one of the most efficient producers of EVs among different cell types (Yeo et al., 2013). Beneficial therapeutic effects of MSC-secretome are mediated at least in part by extracellular vesicles (Nooshabadi et al., 2018). This finding contributed to the development of MSC-based cell-free approach using MSC-derived EVs as therapeutic agents itself or as carrier particles. However, the engineering of EV as drug delivery vehicles requires loading strategies different from those used for liposomes, such as overexpression of proteins and miRNAs into parental cells. To date, most of the proteins selected for overexpression in MSCs have been transcription factors and signaling molecules—c-Myc, GATA-4, HIF-1a, Akt, etc. (Park et al., 2019). Generally, researchers apply viral transduction for efficient genetic modification of MSC. Thus, Akt-exosomes were obtained with adenovirus, CXCR4-exosomes—with a lentiviral vector and GATA-4-exosomes—with a retrovirus encoding GATA-4. However, it should be borne in mind that MSCs are very responsive to environmental changes, rapidly changing their secretion profiles and phenotype upon inflammatory stimuli *in vitro*, including exposure to viral vectors. For our work, we chose recombinant adeno-associated viral vectors, which are well-known for their minimal immunogenicity and excellent safety profile since they are not associated with any known human diseases and have a very low frequency of genome integration. Previously, we have shown that viral transduction has a significant impact on protein cargo of EVs. In the present study, we focused on the microRNA (miRNAs) composition changes in EVs from transduced rat MSCs. Comprehensive studies like this are needed for quality control of EV cargo compositions and the safety and efficacy assessment of EVs from genetically modified MSC before they can be used in clinical applications.

## Materials and Methods

### Cell Isolation

Male Wistar–Kyoto rats (11–12-week-old) were purchased from a Nursery for laboratory animals “Pushchino,” Branch of IBCH, RAS (Pushchino, Russia). Euthanasia was conducted under isoflurane narcosis by secondary cervical spine dislocation in compliance with national and European Union directives and were approved by the Institutional Ethics Board for Animal Care (National Medical Research Center of Cardiology). Rat MSCs were isolated from subcutaneous adipose tissue. Pieces of tissue were minced to a size of 1–2 mm and digested with a mixture of collagenase I (200 U/ml) (Sigma-Aldrich, United States) and dispase (10 U/ml) (Thermo Fisher Scientific Inc., United States) for 30 min at 37°C with continuous stirring. After enzymatic treatment, the cell suspension was centrifugated and filtered through 100-μm nylon cell strainers (BD Bioscience, United States). Cells were seeded in a complete growth medium of 1 g/L of D-glucose DMEM/10% FBS (Gibco, United States) and cultivated under standard conditions (5% CO_2_, 37°C). The next day, the unattached cells were washed out, and the medium was replaced with a fresh one. Then the medium was changed every 2–3 days. Cells were passaged upon reaching 70% confluency using 0.05% trypsin/Versene solution (Paneco, Russia) and subcultured at a 1:4 ratio. Cells were used up including passage 5.

### Adeno-Associated Virus-Vector Preparation and Purification

Rat SCF coding sequence (NM_021843.4) was cloned into a VPK-410 expression vector (Cell Biolabs Inc., United States). The pAAV–hrGFP-expressing vector was purchased from Cell Biolabs, Inc., United States). Packaging vectors VPK-420-DJ (AAB- DJ) and pHelper Vector (No. 340202) (Cell Biolabs, Inc., United States), together with expression plasmids, were used to co-transfect HEK293T.

Cells at 80% confluency were transfected using the calcium phosphate method; 48-h post-transfection cells were harvested by centrifugation and subjected to four freeze–thaw cycles (liquid nitrogen/37°C water bath) and then incubated with 25–50 U/ml of Benzonase (Merck, Germany) at 37°C for 30 min. The solution was cleared by centrifugation at 5,000× *g* (30 min), and a supernatant was used for further purification by an iodixanol density gradient (OptiPrep^TM^; Sigma-Aldrich). The 8 ml of 15% iodixanol layer was underlayered in 35 ml of PA Ultracrimp Tube (03989, Thermo Fisher Scientific Inc., United States) with 6 ml of 25%, 5 ml of 40%, and 5 ml of 60%. Cell lysates were then overlaid onto the gradient, the tube was sealed and centrifuged for 90 min at 280,000× *g*, 16°C in a T-865 rotor (Thermo Fisher Scientific Inc., United States). After centrifugation, the adeno-associated virus vectors (AAV)-containing 40% layer was collected and diluted with the concentration buffer PBS/0.001% Pluronic F-68 (A1288, Applichem).

The AAV product then was concentrated and desalted using the Amicon Ultra-4 (Merck, Germany) centrifugal concentrator devices. Final concentrated product was stored at 4°C.

### AAV Titration

For further analysis, 5 μl of the AAV sample was treated with 3 U of DNaseI (EN0521, Thermo Fisher Scientific Inc., United States) at 37°C for 1 h, and then DNaseI was inactivated at 75°C for 10 min. One unit of Proteinase K (EO0491, Thermo Fisher Scientific Inc., United States) was added, and the solution was incubated at 60°C for 1 h; after which, it was inactivated at 95°C for 10 min.

Quantitative PCR analysis was performed using the Qiagen Rotor-Gene Q system. PCR reactions were carried out in a final volume of 20 μl including qPCRmix-HS (PK145S, Evrogen, Russia) supplemented with 200 nM of forward (GGAACCCCTAGTGATGGAGTT) and reverse primers (CGGCCTCAGTGAGCGA), 100 nM FAM/BHQ probe (CACTCCCTCTCTGCGGCTCG), and diluted plasmid standard or treated AAV sample. The PCR profile was as follows: denaturation step at 95°C for 10 min, 40 cycles of denaturation at 95°C for 15 s, and annealing or extension at 60°C for 1 min.

### Mesenchymal Stem/Stromal Cell Transduction

Rat MSC up to fifth passages were cultured until 70% confluency (corresponds to 1–1.5 × 10^6^ cells). The media was then replaced with DMEM without antibiotics and serum and AAV stock solution at concentration 4.5 ^∗^ 10^11^ gc/ml, MOI 95000).

Cells were incubated for 3 h with mild stirring every 30 min to ensure even cell distribution at 37°C, 5% CO_2_. Afterward, FBS was added, and MSCs were cultivated overnight, then one half of the culture medium was replaced with a fresh one. Cells were passaged 3 days post-transduction.

### Extracellular Vesicle Isolation and Analysis

Extracellular vesicles were isolated from AAV-transduced MSCs conditioned medium at day 7 after transduction or from intact (control) cells. Cells were washed three times from standard culture medium with Hanks balanced salt solution and maintained in DMEM/F-12 with 1% exosome-depleted FBS (System Biosciences, United States) for 48 h. Conditioned media were cleared from cells and debris by serial centrifugations: 400× *g* for 10 min, 2,000× *g* for 30 min. The supernatant (sample volume 36 ml) was then centrifuged at 100,000× *g* and 10°C for 90 min in a SW32 Ti bucket rotor (Beckman Coulter, United States) using ultracentrifuge Optima XE-90 (Beckman Coulter, United States). Pellet was resuspended in DPBS, and the presence of EVs in samples was confirmed by transmission electron microscopy. The protein concentration in EV samples was routinely measured using the Bradford assay.

The size distribution of EVs and their concentration were determined by nanoparticle-tracking analysis (NTA) using NanoSight LM10 HSBF instrument and NTA 2.3 software build 0033 (NanoSight Ltd., United Kingdom). Configuration included 405-nm, 65-mW laser unit with passive temperature readout and high sensitivity camera of EMCCD type. All measurements were performed according to ASTM E2834 (ASTM, 2018), using previously reported camera- and video-processing setups, optimized for EVs (Livshits et al., 2015; Silachev et al., 2019; Evtushenko et al., 2020). Briefly, samples were diluted by particle-free PBS down to the optimal concentration around 1.5 × 10^8^ particles/ml. Fourteen videos at 60 s each were recorded using the fresh portion of a diluted sample for each measurement. Tracks datasets from all videos were merged to obtain joint particle size distribution, mean size and total particle concentration, corrected for the dilution factor.

For TEM imaging, the EV sample was applied to nitrocellulose carbon-coated Cu grids and negatively stained with 2% uranyl acetate solution as described previously (Silachev et al., 2019). The grids were imaged at 80 kV using a JEOL JEM-1011 transmission electron microscope (JEOL, Akishima, Japan) equipped with a digital Orius^TM^ SC1000 W digital camera (Gatan Inc., Pleasanton, CA, United States).

### ELISA Assay

Intact and transduced MSCs were cultivated in a complete growth medium for 48 h.

The conditioned media on the seventh day after transduction were collected, centrifuged to remove floating cells (400× *g*) and debris (2,000× *g*) and stored frozen before analysis. SCF concentrations were measured using SCF Mouse ELISA Kit (Abcam, United States) following the instructions of the manufacturer using Victor^TM^ X3 Multi Label Plate Reader (Perkin Elmer Inc., United States).

### RNA Isolation and Microarray Analysis

Total RNA was isolated using the miRCURY RNA Isolation kit (Exiqon-Qiagen, United States). The total RNA concentration was determined by measuring the absorbance at 260 nm in a spectrophotometer (NanoDrop Technologies, United States). The rat microRNA content was evaluated using the Affymetrix GeneChip^TM^ miRNA Arrays 4.0 kit (Thermo Fisher Scientific, United States) containing probes for 728 mature and 490 rat pro-miRNAs.

RNA was biotin-labeled with a FlashTag^TM^ Biotin HSR RNA Labeling Kit (Thermo Fisher Scientific, United States). Labeled RNA samples were hybridized to the chip in a GeneChip^TM^ Hybridization Oven 645 (Thermo Fisher Scientific, United States) (18 h at 48°C and 60 rpm).

After hybridization, miRNA arrays were washed and stained using the Affymetrix GeneChip^TM^ Hybridization Wash and Stain Kit on GeneChip^TM^ Fluidics Station 450 and were then scanned with the Affymetrix GeneChip^TM^ Scanner 3000 7G (Thermo Fisher Scientific, United States), according to the protocol of the manufacturer. The data were processed using the Transcriptome Analysis Console software 4.0.1 (Thermo Fisher Scientific, United States).

### Microarray Data Processing

CEL-files of raw data were produced with the Transcriptome Analysis Console software (version 4.0.1, Thermo Fisher Scientific, United States). miRNAs that fulfilled the criteria (*p*-value < 0.05, absolute value of fold-change ≥ 2.0) were selected for further analysis.

Hierarchical clustering was performed to build a hierarchy of clusters using the Transcriptome Analysis Console software v4.0.1. The distance metric used between objects is the Euclidean distance. Complete-linkage clustering method was used for measuring the distances between a pair of objects in the two clusters. Results are displayed as a heatmap.

### Network Construction and Analysis

The gene interaction network was built and analyzed using the NetworkX 2.0 package for Python 3. The constructed network consists of “nodes” representing target genes and “edges”—the molecular interactions of their protein products. The resulting network was visualized using the Cytoscape software. All calculations were performed as described earlier (Osmak et al., 2020).

### Data Analysis

Data displayed in diagrams are either as mean ± SD or as representative single experiments. NTA data are presented as mean ± 95% confidence interval, calculated from individual measurements using Student’s *t*-distribution.

Unpaired two-tailed Student’s *t*-test was used to compare groups for RNA content. A value of *p* > 0.05 equals not significant, ≤ 0.05^∗^, ≤ 0.01^∗∗^, ≤ 0.001^∗∗∗^. Two-way ANOVA was used for microarrays data *p*-value calculation.

## Results

### Morphological Observation of Extracellular Vesicles From Mesenchymal Stem/Stromal Cells

**FIGURE 1:**
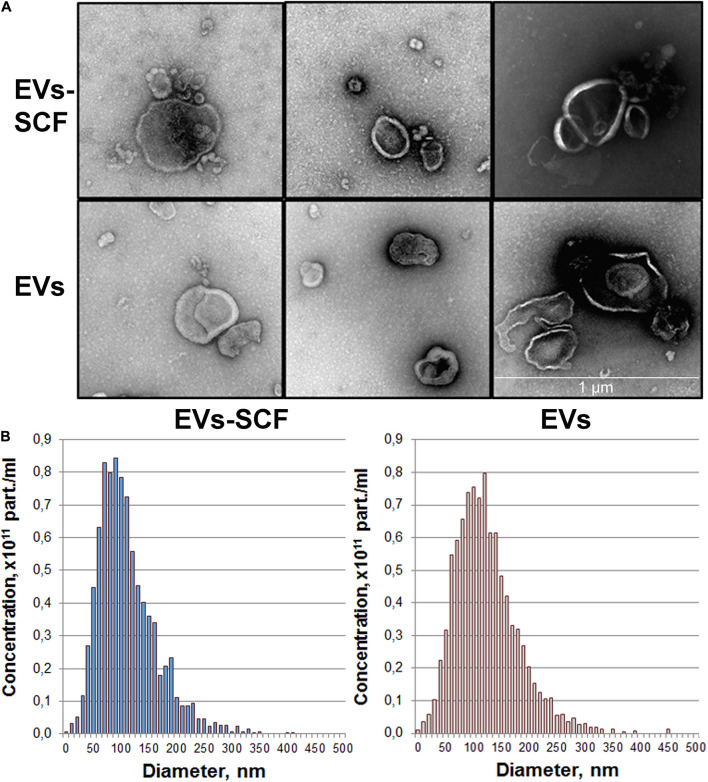
Quality control of extracellular vesicles (EVs) secreted by intact or AAV-transduced mesenchymal stem/stromal cells. **(A)** Representative transmission electron microscopy images of pooled EVs isolated by ultracentrifugation. Scale bar = 1 μm. **(B)** Nanoparticle tracking analysis of MSC extracellular vesicles. Each histogram represents the frequency of EVs in a particular size, ranging from 0 to 500 nm.

Rat adipose-tissue-derived mesenchymal cells were transduced with highly effective AAV-DJ viral vector, encoding rat KITLG gene or Stem Cell Factor (SCF). For infection efficiency monitoring, we used GFP-transduced the MSC. At day 5 postinfection, MSC culture media were replaced by media supplemented with exosome-depleted FBS, and 48 h after, media

were collected for extracellular vesicles isolation. Purified EVs were characterized phenotypically by TEM and NTA analysis. TEM confirmed the presence of intact vesicular structures with classical morphology such as clearly discernible lipid bilayers and “cup shape” (Figure 1A). We did not observe visible differences between vesicles from transduced (SCF) or intact (control) cells. NTA analysis revealed that cultured MSC-released vesicles with a mean size of 120 nm (Figure 1B), which is within the size range for microvesicles (0.1–1.0 μm) and overall profile exhibits a size distribution of particles mostly less than 200 nm. NTA also revealed that the particle concentration, mean particle size (Table 1), and size distribution (Figure 1B) were not significantly different between transduced or intact MSCs. However, SCF vesicles demonstrate a clear tendency to smaller size and less average protein content, but total RNA content significantly distinguishes between groups (Table 1).

**TABLE 1:** Quantitative characterization of extracellular vesicles (EVs).

Sample	Mean diameter, nm	Concentration, × 10^11^ particles/ml	The average yield of protein (μg) per 150 ml of medium	The average yield of RNA (ng) per 1 ml of medium
EVs	129 ± 9	9.6 ± 1.4	37.74 ± 0.9	29.27 ± 15.82
EVs-stem cell factor (SCF)	116 ± 17	8.9 ± 1.5	31.36 ± 21.02	17.3 ± 9.8* (*p* = 0.028)

* statistically significant.

### Analysis of miRNA Distribution Between Extracellular Vesicles and Parental Cells

**FIGURE 2:**
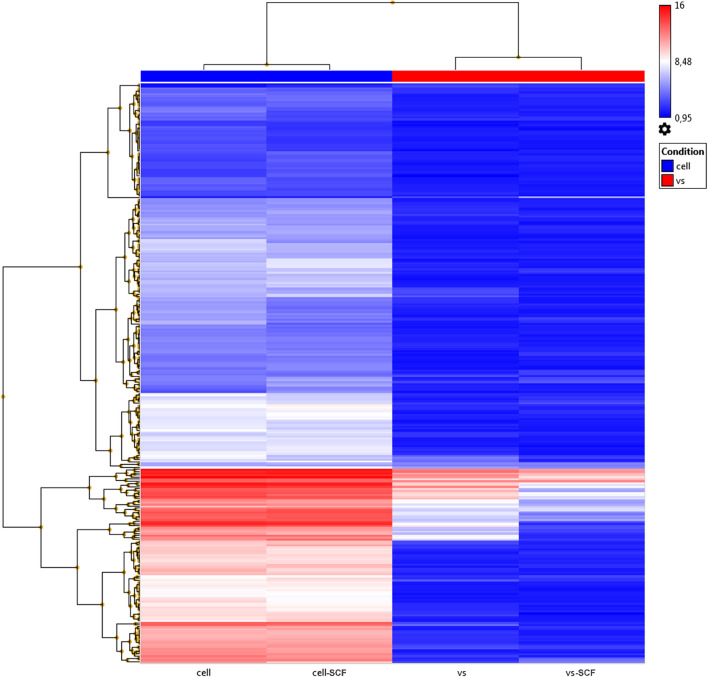
Hierarchical clustering dendrograms of differentially expressed miRNA expression patterns in EVs and cell samples obtained by the GeneChip^TM^ miRNA Arrays 4.0.1.

Previously, we examined changes in protein content by mass spectrometry (Zubkova et al., 2019; Dergilev et al., 2020) to determine if rAAV infection could induced alteration in MSC EV cargoes and did not find significant differences between EVs from intact or transduced MSC (less than 5% of unique proteins). In the present study, we focused on microRNA content of EVs. We analyzed the miRNA content of MSC-derived exosomes using the GeneChip^TM^ miRNA Arrays 4.0 kit. The number of miRNAs detected in cells above background (DABG) was 1,250. Most miRNA were equally present in both the EVs and cells. However, we identified about 300 miRNAs significantly downregulated in EVs compared with cells with threshold settings *p*-of value ≤ 0.05 and fold change ≥ 2. Only seven to nine miRNAs with unknown targets were enriched in EVs but insufficiently significantly. To analyze the enriched pathways targeted by downregulated miRNAs, we used KEGG or WikiPathways databases and g:Profiler—the web server for functional enrichment analysis. Search in Gene Ontology (GO) biological process categories revealed among the top terms “cellular response to oxygen-containing compound” (GO:1901701), “cell population proliferation” (GO: 0008283), and others and target genes (192) were significantly enriched in a total of 56 KEGG pathways including the “FoxO signaling pathway” (KEGG:04068). According to the WikiPathways database, targets were significantly enriched in 11 pathways (Table 2). Overall, we found that miRNAs were more abundant in parental cells (Figure 2). A heatmap visualized clustering between the cellular and the EV miRNAs (Figure 2). Within this heatmap, clear division in the pattern of miRNA expression is evident between the cells and extracellular vesicle samples.

**TABLE 2:** Pathway enrichment analysis of downregulated miRNA target gene set in the WikiPathways database.

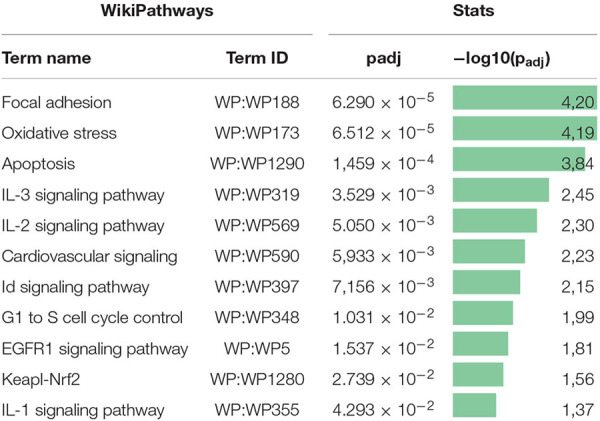

### Comparison of miRNA Content Between Extracellular Vesicles From Intact or AAV-Transduced Mesenchymal Stem/Stromal Cells

**FIGURE 3:**
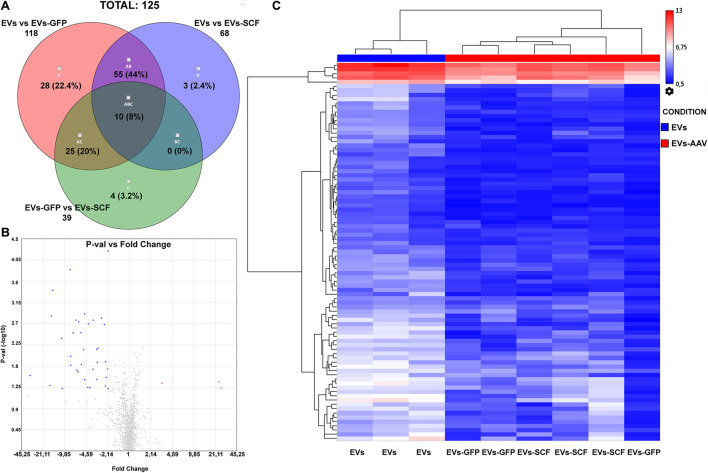
**(A)** Venn diagram summarizes the number of miRNAs that are differentially expressed between EVs from stem cell factor (SCF) or green fluorescent protein (GFP)-transduced MSCs or collected from intact cells. **(B)** Volcano plot (*p*-value vs. fold change (FC), FC ≤ –2 and FC ≥ 2) of EV miRNA from GFP-transduced MSC over EVs from SCF-transduced MSCs. Upregulated (red) and downregulated (blue) miRNA with twofold change are shown; the plot was generated using the TAC 4.0 software (Thermo Fisher Scientific, United States). **(C)** Hierarchical clustering of miRNA (two-way ANOVA, *p*-value < 0.05) from EV samples (from intact or AAV-infected MSCs). MiRNA levels are shown as a heat map. Complete-linkage clustering method.

The differentially expressed miRNAs between exosomes from AAV-transduced or intact (control) cells were analyzed similarly as the distribution of miRNAs between parental cells and EVs. miRNA microarray analysis was performed with the following settings: *p*-value ≤ 0.05 and fold change absolute value ≥ 2. The Venn diagram depicts the overlapping differentially expressed miRNAs between all samples. According to our results, the difference between EVs from SCF or GFP-transduced MSCs was negligible—3.4% (Figure 3A) and very few miRNAs, which were above fivefold upregulated or downregulated (Figure 3B). That indicates rather intrinsic changes in MSC after viral transduction than the transgene effect by itself. Hierarchical clustering of the detected miRNAs clearly identified EVs from transfected MSCs (SCF and GFP together) and control MSCs as two separate groups (Figure 3C). We uncovered a total of 91 differentially expressed miRNAs and identified two miRNAs significantly upregulated in AAV-transduced MSCs. One of them—rno-miR-344a-2—was previously associated with aging and regulation of a-SMA (Wang et al., 2015) and the other—rno-miR-208a-3p—mediates the myocardial endoglin expression that led to increased myocardial fibrosis (Wang et al., 2014). Eighty-nine miRNAs were downregulated in both EVs from SCF- or GFP-MSC compared with EVs from intact MSCs. Network analysis of genes that are targeted by these downregulated miRNAs were plotted using the String database (Supplementary Material 1). Moreover, we performed pathway enrichment analysis of these genes and found that they were enriched in the categories of PI3-Act and FoxO-signaling pathways (Supplementary Material 2).

### miRNA-Based Network Analysis

**FIGURE 4:**
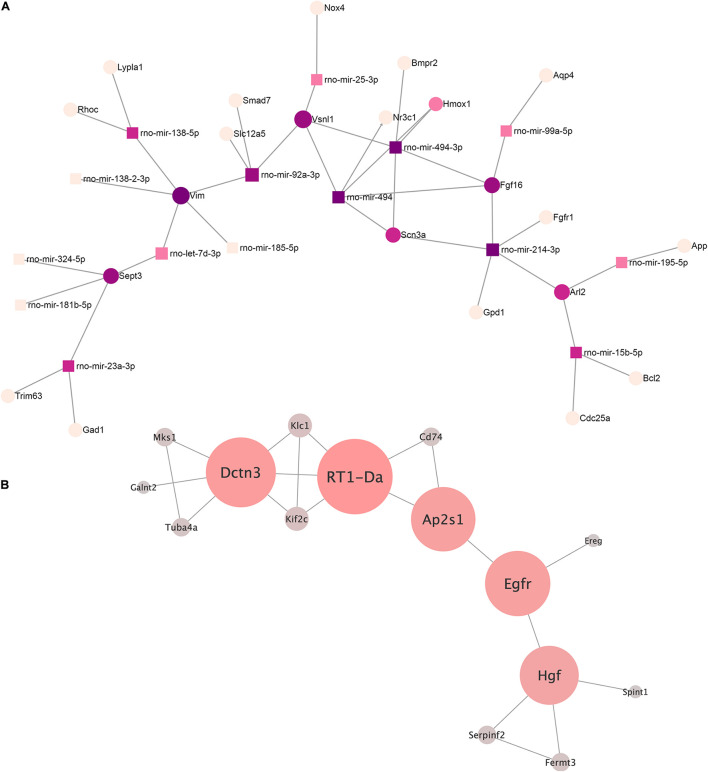
**(A)** The microRNA–target gene interaction network of the differentially expressed miRNA (log2 fold change ≥ 2.0, ≤ –2.0) was constructed using the miRNet 2.0 network-based visual analytic tool. Nodes indicated by rectangles represent signature miRNAs. Nodes indicated by circles represent target genes including violet nodes representing “hub” genes, regulated more than four miRNAs. **(B)** Network of rno-miRNA-128-3p targeted gene intermolecular interactions. The color gradient from gray to red and the size shows the centrality of the nodes.

We built an interaction network of genes regulated by miRNAs decreased in EVs from AAV-transduced rat MSC using miRNet 2.0—a network-based visual analytic tool (Chang et al., 2020). The algorithm divided miRNAs into 13 separate networks, most of which consist of one or two miRNAs. For further analysis, we chose the largest network (Figure 4A) and the network of rno-mir-128-3p target genes, each of which had at least one edge over another target gene (Figure 4B). The target genes of other microRNAs did not form a network like this.

We identified four “hub” genes: Vim, Sept3, FGF16, and Vsnl1. Vim, Sept3, and Vsnl1 are involved in the regulation of cellular migration, which is consistent with our previous data on the regulation of c-kit cell migration by EVs (Zubkova et al., 2019).

We analyzed the network of rno-mir-128-3p target genes using our previously described method (Osmak et al., 2020) in order to better understand its possible regulatory effects. Target genes of our miRNAs were taken as network nodes according to the miRTarBase and TarBase databases. Any two nod genes were considered linked if their protein products have an interaction score greater than 0.9 (String interaction score) according to the String database. A total of 15 such genes were identified.

The key genes for this network were extracted based on our previously developed algorithm (Osmak et al., 2020). The following genes were identified: RT1-Da, Dctn3, Egfr, Ap2s1, and Hgf (listed by decreasing centrality). To clarify their functional role, a reactome overrepresentation analysis was performed. It turned out that RT1-Da, Dctn3, and Ap2s1 were enriched in the signaling pathway “MHC class II antigen presentation” (*p* = 0.0058), and Egfr and Ap2s1 were enriched in the “L1CAM interactions” (*p* = 0.023). The rest of the pathways are overrepresented by no more than one gene from our key gene set, and therefore, we did not consider it for further analysis.

## Discussion

Mesenchymal stromal cells rapidly gained popularity in clinical applications due to their unique features. MSCs have immunosuppressive and anti-inflammatory properties and can be isolated in sufficient quantities from a variety of tissues and easily expanded *in vitro*. However, it became apparent that most of the MSC therapeutic effects can be recapitulated with administration of cell-free MSC-conditioned media that contains extracellular vesicles among other moieties (Gnecchi et al., 2005; Timmers et al., 2011). Moreover, MSCs are considered one of the most prolific EV-producer cell types (Yeo et al., 2013). The therapeutic use of MSC-derived EVs especially with designed cargo and/or surface might be more practical than attempts to repair damaged tissues by the use of intact MSCs. The advantage of EVs as a means of intercellular communication has been further exploited to deliver therapeutic signals such as proteins and miRNAs. MSCs are often genetically engineered with viral vectors to overexpress specific miRNAs or proteins that are then incorporated into the EV cargo (Xin et al., 2013; Yu et al., 2015; O’Brien et al., 2018; Sun et al., 2020; Zilun et al., 2020). Although this is often ignored, viral transduction does not go unnoticed by cells; for instance, AAV infection may elicit DNA-damage-response signaling pathways (Winocour et al., 1988; Saudan et al., 2000; Fragkos et al., 2009). It is suggested that the AAV-induced DNA damage response is provoked by inverted terminal repeats (ITR), which are present in both the wild-type virus and recombinant AAV vectors (Raj et al., 2001). Being aware that MSCs are very sensitive to their microenvironment and given the importance of miRNAs in regulating multiple cellular responses, we aimed at characterizing the expression profile of miRNAs in EVs from MSCs. We investigated the impact of transduction, with that widely used in experimental and clinic studies, recombinant adeno-associated viral vector, on miRNome of EVs. Recent works raise the question about changes in EV molecular cargo depending on the manipulation with the parent MSC—ionizing radiation (Abramowicz et al., 2020), treatment with inflammatory cytokines (Huang et al., 2019), hypoxia (Salomon et al., 2013), and aging (Fafián-Labora et al., 2017). All these studies showed that microenvironmental stresses lead to substantial differences in miRNome or proteome of extracellular vesicles, but the data suggesting the impact of transduction with viral vectors on EVs cargo are very limited. Nevertheless, we suggest that comprehensive understanding of the alteration in EV cargo after transduction with viral vectors is crucial for both: correct interpretation of experimental study results and future development of combined strategies for gene-cell and cell-free therapy.

Our previous studies had shown that rat MSC transduction with AAV viral vector (Dj serotype) leads to the mRNA of stress marker PAI2 expression in cells and subtle changes in the vesicular cargo protein composition (Zubkova et al., 2019, 2021). Using mass-spectrometry analysis, we revealed unexpected enrichment of glycolysis/gluconeogenesis enzymes, proteins associated with collagen synthesis and viral life circle, as well as antioxidant enzymes (SOD2, PRDX5, PRDX6) in the EVs isolated from AAV-transduced MSCs (Zubkova et al., 2019). In the present study, we analyzed changes in miRNA content in EVs from intact or AAV-transduced MSCs using intracellular green fluorescent protein (GFP) and secreted stem cell factor (SCF) as transgenes. In accordance with our previous studies, we did not reveal the differences in the protein quantity in EVs from transduced or non-transduced cells. Using nanoparticle tracking assay as a more accurate method for EV quantification, we confirmed that there were no significant differences in the EV concentration or size distribution. At the same time, total RNA concentration in EVs from AAV-transduced cells was significantly less than in non-transduced MSCs. EV samples were characterized by TEM and met the standard criteria of vesicles morphologically.

Several studies comparing the miRNA content between parental MSCs and EVs have shown a selective miRNA sorting in EVs, although the underlying mechanism has remained unclear until now (Qiu et al., 2018). EV biogenesis is closely related to the cellular endocytosis pathway.

The ESCRT-endosomal-sorting complex required for transport family has been shown to play a key role in cargo loading and exosome biogenesis, but there are also ESCRT-independent pathways in cargo sorting. The pathways may not be entirely separated. In fact, they may work synergistically, and different subpopulations of EVs could depend on different machineries (Qiu et al., 2021). Currently, 604 miRNAs have been identified in EVs from human adipose tissue-derived MSCs (Alonso-Alonso et al., 2021). However, there is a lack of a consensus miRNA signature among MSC-EVs from different studies (Qiu et al., 2018).

Our microarray analysis showed substantial differences in miRNA expression in EVs compared with their donor cells. We identified about 300 miRNAs significantly reduced in EVs compared with parental cells. This ensured that miRNA expression profile in EVs differs from parental cell, and miRNA in EVs not just randomly reflects cellular miRNA content.

Pathway enrichment analysis of validated genes targeted by downregulated miRNAs had shown that EVs contain less miRNA involved in the regulation of key pathways—proliferation, apoptosis, and interleukin signaling, than the whole cells.

Hierarchical clustering of differentially expressed miRNAs from EVs of intact and transduced MSCs revealed a high expression profile similarity between the AAV-treated cells, which were significantly different from the untreated control. Overall, about 10% of miRNAs were decreased in EVs from transduced cells regardless of the transgene used. We hypothesized that this could be attributed to the stressor effect of AAV-vector transduction on donor cells.

Similar data were obtained by the Huang group, which showed that exosomes from MSCs treated with TNFα and IL6 had more downregulated, differentially expressed miRNAs, particularly angiogenesis-related miRNAs compared with control (Huang et al., 2019). In another work, among 25 miRNAs that were differentially expressed in TNF-treated MSCs, 20 miRNAs were downregulated, while only 5 miRNAs were upregulated (Fayyad-Kazan et al., 2017).

Gene targets of downregulated miRNAs form a complex interaction network that include several transcription factors: FoxO3, Myc, Hif1a, etc. (Supplementary Material 1). This allows us to suggest that transduction with AAV can alter the abilities of the EVs to regulate a range of signaling pathways and homeostasis network. An advanced bioinformatic analyses of downregulated miRNA targets revealed four “hub” genes—Vim, Sept3, Fgf16, and Vsnl1, for each of which more than four regulatory miRNAs were simultaneously decreased. These proteins and the key targets of rno-miRNA-128-3p (RT1-Da, Dctn3, Egfr, Ap2s1, Hgf), participates in the PI3K–Akt signaling pathway, vesicular trafficking machinery, cell growth, and tissue repair. It should be mentioned that until now, very few potential targets of rat miRNAs were validated.

Our findings about the miRNA cargo of EVs are in accordance with our previous studies, which also showed changes in donor cells itself (proliferation rate decrease, enhanced expression of PAI-2) and EV protein content upon AAV-vector transduction. In combination with our previous data, these findings reflect the overall picture of the stress response to AAV-infection in MSCs that leads to significant changes in vesicular cargo content (both protein and miRNA).

Our paper not only provides a detailed characterization of the AAV-induced changes in EV miRNA expression profile but also increases our knowledge about parental cell condition-dependent variability of the EVs. This study contributes to better understanding of the stress influence on EV diversity. It also highlights that the indirect effects of any *in vitro* manipulation with cells may have a great impact on EVs as a delivery vehicle for experimental therapy and clinical application. Studies like ours, evaluating the changes in EV cargo depending on the manipulation with the parent MSC, will allow to select the optimal strategies for EV loading for cell-free therapy.

## Data Availability Statement

The datasets presented in this study can be found in online repositories. The names of the repository/repositories and accession number(s) can be found below: ArrayExpress, E-MTAB-10858.

## Ethics Statement

The animal study was reviewed and approved by the Institutional Ethics Board for Animal Care (National Medical Research Center of Cardiology.

## Author Contributions

EZ, MM, and YP conceptualized the study. EZ, IB, EE, and MM were in charge of the data curation. EZ, IB, EE, PK, AM, and GO investigated the study and developed the methodology. EZ wrote the manuscript. YP revised the manuscript and acquired funding. All authors have read and agreed to the published version of the manuscript.

## Conflict of Interest

PK was employed by Genomed Ltd. The remaining authors declare that the research was conducted in the absence of any commercial or financial relationships that could be construed as a potential conflict of interest.

## Publisher’s Note

All claims expressed in this article are solely those of the authors and do not necessarily represent those of their affiliated organizations, or those of the publisher, the editors and the reviewers. Any product that may be evaluated in this article, or claim that may be made by its manufacturer, is not guaranteed or endorsed by the publisher.

## Supplementary Material

Supplementary Material 1 Network analysis of genes targeted by miRNAs downregulated in EVs from AAV-viral vector transduced MSC.

Supplementary Material 2 Enrichment analysis of genes targeted by miRNAs downregulated in EVs from AAV-viral vector transduced MSC.

## References

[B1] Abramowicz A. Łabaj W. Mika J. Szołtysek K. Ślęzak-Prochazka I. Mielańczyk Ł (2020). MicroRNA profile of exosomes and parental cells is differently affected by ionizing radiation. *Radiat. Res.* 194 133–142. 10.1667/RADE-20-00007 32383628

[B2] Alonso-Alonso M. L. García-Posadas L. Diebold Y. (2021). Extracellular vesicles from human adipose-derived mesenchymal stem cells: a review of common cargos. *Stem Cell Rev. Rep.* 10.1007/s12015-021-10155-5 33904115 PMC8942954

[B3] ASTM (2018). *Standard Guide for Measurement of Particle Size Distribution of Nanomaterials in Suspension by Nanoparticle Tracking Analysis (NTA).* West Conshohocken, PA: ASTM International.

[B4] Chang L. Zhou G. Soufan O. Xia J. (2020). miRNet 2.0: network-based visual analytics for miRNA functional analysis and systems biology. *Nucleic Acids Res.* 48 244–251. 10.1093/nar/gkaa467 32484539 PMC7319552

[B5] Dergilev K. V. Shevchenko E. K. Tsokolaeva Z. I. Beloglazova I. B. Zubkova E. S. Boldyreva M. A. (2020). Cell sheet comprised of mesenchymal stromal cells overexpressing stem cell factor promotes epicardium activation and heart function improvement in a rat model of myocardium infarction. *Int. J. Mol. Sci.* 21:9603. 10.3390/ijms21249603 33339427 PMC7766731

[B6] Evtushenko E. G. Bagrov D. V. Lazarev V. N. Livshits M. A. Khomyakova E. (2020). Adsorption of extracellular vesicles onto the tube walls during storage in solution. *PLoS One* 15:e0243738. 10.1371/journal.pone.0243738 33370319 PMC7769454

[B7] Fafián-Labora J. Lesende-Rodriguez I. Fernández-Pernas P. Sangiao-Alvarellos S. Monserrat L. Arntz O. J. (2017). Effect of age on pro-inflammatory miRNAs contained in mesenchymal stem cell-derived extracellular vesicles. *Sci. Rep.* 7:43923. 10.1038/srep43923 28262816 PMC5338265

[B8] Fayyad-Kazan H. Fayyad-Kazan M. Badran B. Bron D. Lagneaux L. Najar M. (2017). Study of the microRNA expression profile of foreskin derived mesenchymal stromal cells following inflammation priming. *J. Transl. Med.* 15:10. 10.1186/s12967-016-1106-3 28086811 PMC5237315

[B9] Fragkos M. Jurvansuu J. Beard P. (2009). H2AX is required for cell cycle arrest via the p53/p21 pathway. *Mol. Cell. Biol.* 29 2828–2840. 10.1128/MCB.01830-08 19273588 PMC2682023

[B10] Gnecchi M. He H. Liang O. D. Melo L. G. Morello F. Mu H. (2005). Paracrine action accounts for marked protection of ischemic heart by Akt-modified mesenchymal stem cells. *Nat. Med.* 11 367–368. 15812508 10.1038/nm0405-367

[B11] Huang C. Luo W. F. Ye Y. F. Lin L. Wang Z. Luo M. H. (2019). Characterization of inflammatory factor-induced changes in mesenchymal stem cell exosomes and sequencing analysis of exosomal microRNAs. *World J. Stem Cells* 11 859–890. 10.4252/wjsc.v11.i10.859 31692888 PMC6828590

[B12] Livshits M. A. Khomyakova E. Evtushenko E. G. Lazarev V. N. Kulemin N. A. Semina S. E. (2015). Isolation of exosomes by differential centrifugation: theoretical analysis of a commonly used protocol. *Sci. Rep.* 5:17319. 10.1038/srep17319 26616523 PMC4663484

[B13] Nooshabadi V. T. Mardpour S. Yousefi-Ahmadipour A. Allahverdi A. Izadpanah M. Daneshimehr F. (2018). The extracellular vesicles-derived from mesenchymal stromal cells: a new therapeutic option in regenerative medicine. *J. Cell Biochem.* 119 8048–8073. 10.1002/jcb.26726 29377241

[B14] O’Brien K. P. Khan S. Gilligan K. E. Zafar H. Lalor P. Glynn C. (2018). Employing mesenchymal stem cells to support tumor-targeted delivery of extracellular vesicle (EV)-encapsulated microRNA-379. *Oncogene* 37 2137–2149. 10.1038/s41388-017-0116-9 29367765

[B15] Osmak G. Kiselev I. Baulina N. Favorova O. (2020). From miRNA target gene network to miRNA function: miR-375 might regulate apoptosis and actin dynamics in the heart muscle via Rho-GTPases-dependent pathways. *Int. J. Mol. Sci.* 21:9670. 10.3390/ijms21249670 33352947 PMC7765785

[B16] Park K. S. Bandeira E. Shelke G. V. Lässer C. Lötvall J. (2019). Enhancement of therapeutic potential of mesenchymal stem cell-derived extracellular vesicles. *Stem Cell Res. Ther.* 10:288. 10.1186/s13287-019-1398-3 31547882 PMC6757418

[B17] Qiu G. Zheng G. Ge M. Wang J. Huang R. Shu Q. (2018). Mesenchymal stem cell-derived extracellular vesicles affect disease outcomes via transfer of microRNAs. *Stem Cell Res. Ther.* 9:320. 10.1186/s13287-018-1069-9 30463593 PMC6249826

[B18] Qiu Y. Li P. Zhang Z. Wu M. (2021). Insights into exosomal non-coding RNAs sorting mechanism and clinical application. *Front. Oncol.* 11:664904. 10.3389/fonc.2021.66490 PMC8111219 33987099

[B19] Raj K. Ogston P. Beard P. (2001). Virus-mediated killing of cells that lack p53 activity. *Nature* 412 914–917. 10.1038/35091082 11528480

[B20] Salomon C. Ryan J. Sobrevia L. Kobayashi M. Ashman K. Mitchell M. (2013). Exosomal signaling during hypoxia mediates microvascular endothelial cell migration and vasculogenesis. *PLoS One* 8:e68451. 10.1371/journal.pone.0068451 23861904 PMC3704530

[B21] Saudan P. Vlach J. Beard P. (2000). Inhibition of S-phase progression by adeno-associated virus Rep78 protein is mediated by hypophosphorylated pRb. *EMBO J.* 19 4351–4361. 10.1093/emboj/19.16.4351 10944118 PMC302033

[B22] Silachev D. N. Goryunov K. V. Shpilyuk M. A. Beznoschenko O. S. Morozova N. Y. Kraevaya E. E. (2019). Effect of MSCs and MSC-derived extracellular vesicles on human blood coagulation. *Cells* 8:258. 10.3390/cells8030258 30893822 PMC6468445

[B23] Sun L. Zhu W. Zhao P. Zhang J. Lu Y. Zhu Y. (2020). Down-regulated exosomal MicroRNA-221 - 3p derived from senescent mesenchymal stem cells impairs heart repair. *Front. Cell Dev. Biol.* 8:263. 10.3389/fcell.2020.00263 32432109 PMC7214920

[B24] Timmers L. Lim S. K. Hoefer I. E. Arslan F. Lai R. C. van Oorschot A. A. M. (2011). Human mesenchymal stem cell-conditioned medium improves cardiac function following myocardial infarction. *Stem Cell Res.* 6 206–214. 10.1016/j.scr.2011.01.001 21419744

[B25] Wang B. W. Wu G. J. Cheng W. P. Shyu K. G. (2014). MicroRNA-208a increases myocardial fibrosis via endoglin in volume overloading heart. *PLoS One* 9:e84188. 10.1371/journal.pone.0084188 24392114 PMC3879305

[B26] Wang Y. Fu B. Sun X. Li D. Huang Q. Zhao W. (2015). Differentially expressed microRNAs in bone marrow mesenchymal stem cell-derived microvesicles in young and older rats and their effect on tumor growth factor-β1-mediated epithelial-mesenchymal transition in HK2 cells. *Stem Cell Res. Ther.* 6:185. 10.1186/s13287-015-0179-x 26415502 PMC4587922

[B27] Winocour E. Callaham M. F. Huberman E. (1988). Perturbation of the cell cycle by adeno-associated virus. *Virology* 167 393–399. 2849233

[B28] Xin H. Li Y. Liu Z. Wang X. Shang X. Cui Y. (2013). MiR-133b promotes neural plasticity and functional recovery after treatment of stroke with multipotent mesenchymal stromal cells in rats via transfer of exosome-enriched extracellular particles. *Stem Cells* 31 2737–2746. 23630198 10.1002/stem.1409 PMC3788061

[B29] Yeo R. W. Lai R. C. Zhang B. Tan S. S. Yin Y. Teh B. J. (2013). Mesenchymal stem cell: an efficient mass producer of exosomes for drug delivery. *Adv. Drug Deliv. Rev.* 65 336–341. 10.1016/j.addr.2012.07.001 22780955

[B30] Yu B. Kim H. W. Gong M. Wang J. Millard R. W. Wang Y. (2015). Exosomes secreted from GATA-4 overexpressing mesenchymal stem cells serve as a reservoir of anti-apoptotic microRNAs for cardioprotection. *Int. J. Cardiol.* 182 349–360. 10.1016/j.ijcard.2014.12.043 25590961 PMC4382384

[B31] Zilun W. Shuaihua Q. Jinxuan Z. Yihai L. Qiaoling L. Zhonghai W. (2020). Corrigendum to “miRNA-181a over-expression in mesenchymal stem cell-derived exosomes influenced inflammatory response after myocardial ischemia-reperfusion injury. *Life Sci.* 256:118045. 10.1016/j.lfs.2020.118045 32646681

[B32] Zubkova E. S. Beloglazova I. B. Evtushenko E. G. Kopylov A. T. Shevchenko E. K. Dergilev K. V. (2019). Application of adeno-associated virus vectors for engineering scf-containing extracellular vesicles of mesenchymal stromal cells. *Bull. Exp. Biol. Med.* 166 527–534. 10.1007/s10517-019-04387-2 30793234

[B33] Zubkova E. S. Beloglazova I. B. Ratner E. I. Dyikanov D. T. Dergilev K. V. Menshikov M. Y. (2021). Transduction of rat and human adipose-tissue derived mesenchymal stromal cells by adeno-associated viral vector serotype DJ. *Biol. Open* 19:bio058461. 10.1242/bio.058461 34494647 PMC8443863

