# Supplementary figures and images for: Analysis of MicroRNA Profile Alterations in Extracellular Vesicles From Mesenchymal Stromal Cells Overexpressing Stem Cell Factor
Source: Front Cell Dev Biol. 2021 Nov 15;9:754025. doi: 10.3389/fcell.2021.754025 (PMC8634878; doi:10.3389/fcell.2021.754025)

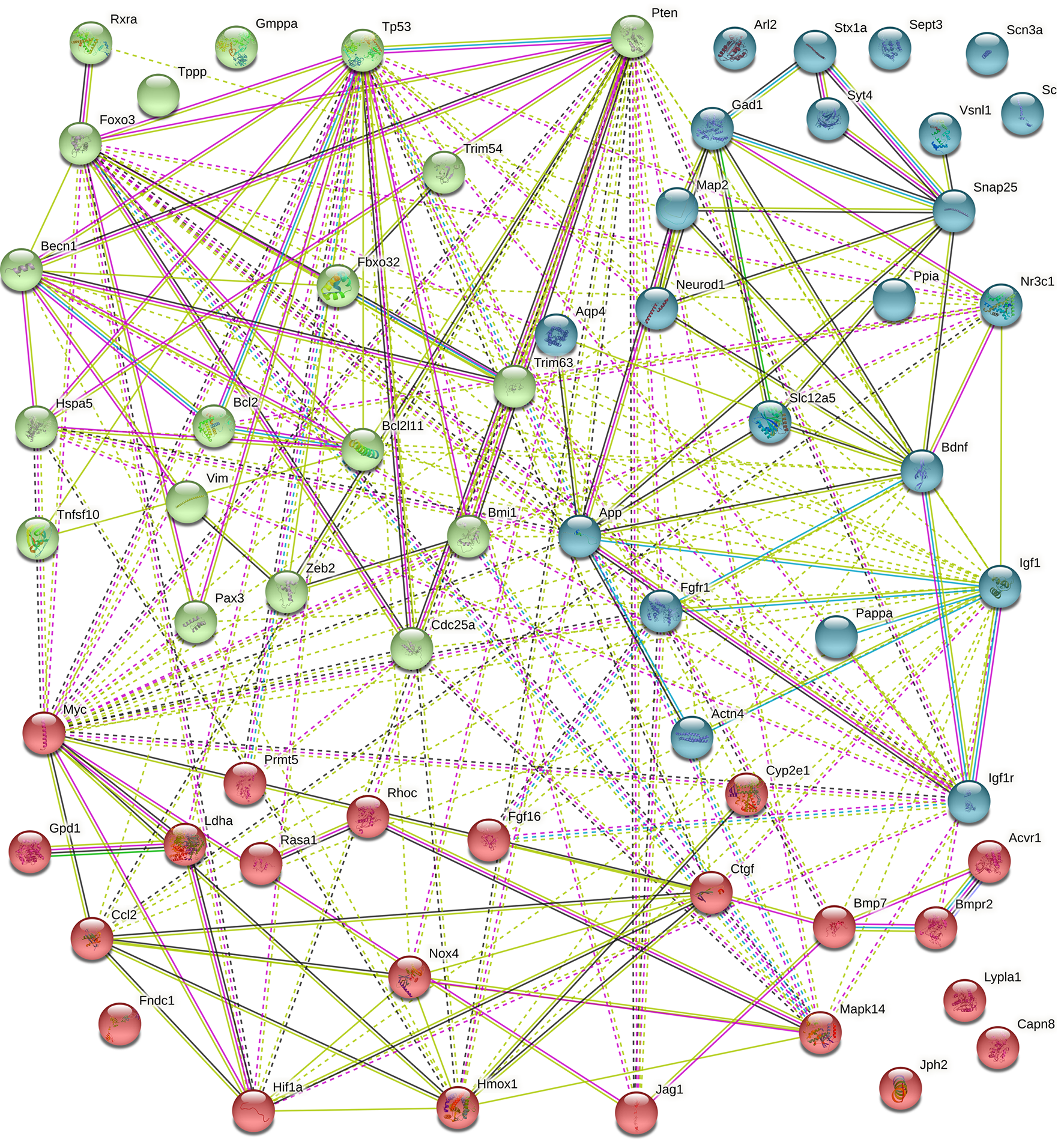

Supplement: Supplementary Material 1 — Network analysis of genes targeted by miRNAs downregulated in EVs from AAV-viral vector transduced MSC. [file Image_1.TIF]

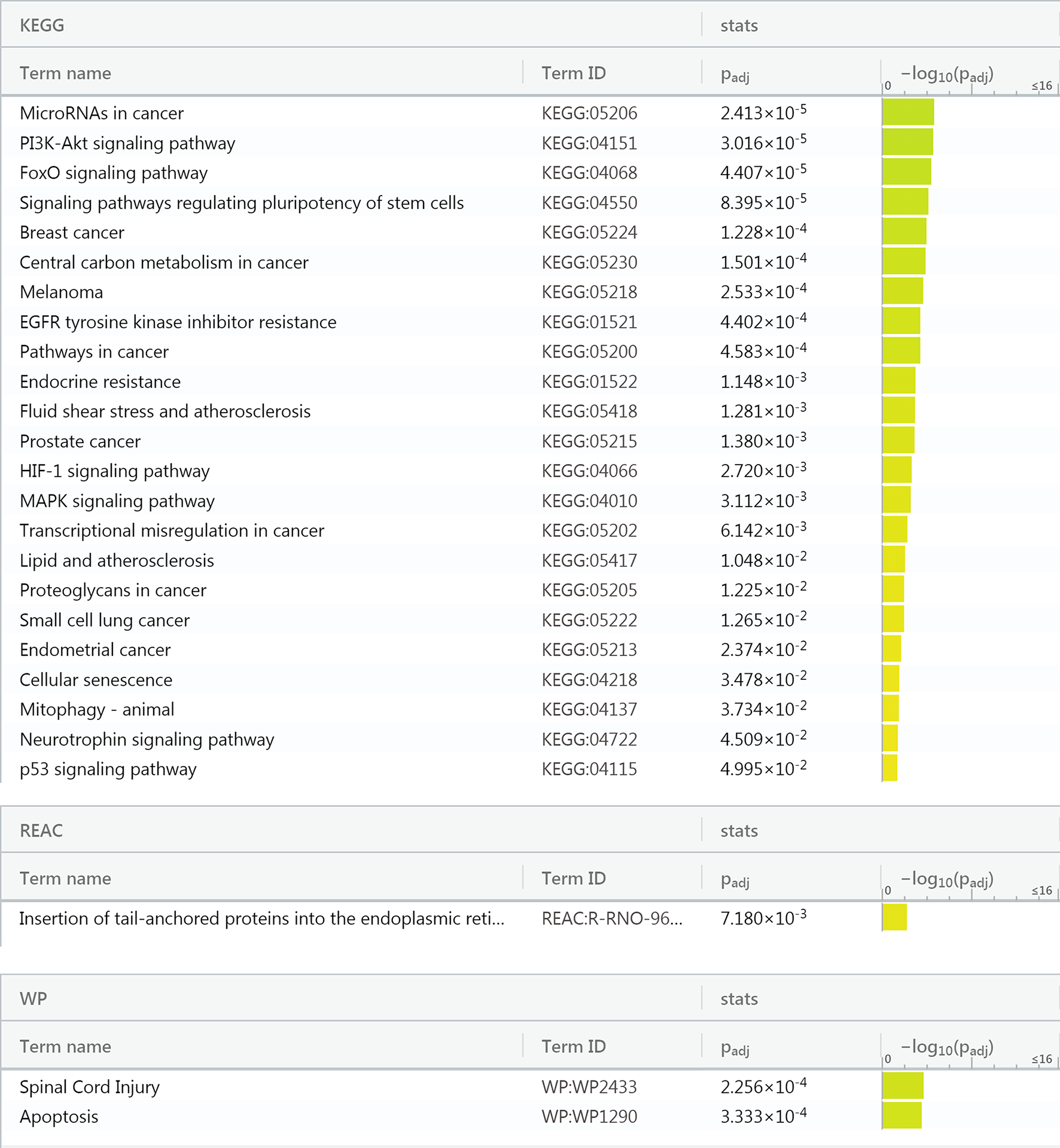

Supplement: Supplementary Material 2 — Enrichment analysis of genes targeted by miRNAs downregulated in EVs from AAV-viral vector transduced MSC. [file Image_2.TIF]
